# Supplementary material for: Using publicly available satellite imagery and deep learning to understand economic well-being in Africa
Source: Nat Commun. 2020 May 22;11:2583. doi: 10.1038/s41467-020-16185-w (PMC7244551; doi:10.1038/s41467-020-16185-w)
Supplement: Supplementary file 1 — Supplementary Information [file 41467_2020_16185_MOESM1_ESM.pdf]

Yeh et al "Using publicly available satellite imagery and deep learning to understand economic well-being in Africa"

Supplementary Information

Supplementary Note 1: Simulation showing difficulty of predicting deltas Suppose there are 2 years of data on 1000 households indexed by i, each falling into village c. Each village has n_c households ranging from 1 to 50. Outcomes y_{ic} are related to observable features x_{ic}, and are generated as follows:

$$y_{ic1} = x_{ic1} + \epsilon_{ic1} \tag{2}$$

with $x_{ic1} \sim N(0, 0.9)$ and $\epsilon_{ic1} \sim N(0, 0.6)$, with the standard deviations of the two terms chosen to generate the variation we see in our ground data and the cross-sectional predictive performance that matches what we see in our deep learning experiments ($r^2 = 0.6 - 0.7$).

Features change over time:

$$x_{ic2} = x_{ic1} + dx_c \tag{3}$$

i.e. features in year 2 are features in year 1 + a village-specific change (dx_c), with $dx_c \sim N(0.08, 0.25)$ as observed in our data.

$$y_{ic2} = x_{ic2} + \epsilon_{ic2} \tag{4}$$

with $\epsilon_{ic2} \sim N(0, 0.6)$ and $cor(\epsilon_{ic1}, \epsilon_{ic2}) = 0$. Changes in household wealth over time are then $dy_{ic} = y_{ic2} - y_{ic1}$. To mirror our survey data, where we observe villages but not households, we then collapse all variables to the village level averages. Then we estimate linear regressions relating outcomes to features in cross section in years 1 and 2:

$$y_{c1} = \beta_1 x_{c1} + \varepsilon_{c1} \tag{5}$$

$$y_{c2} = \beta_2 x_{c2} + \varepsilon_{c2} \tag{6}$$

and over time:

$$dy_c = \beta_3 dx_c + \varepsilon_c \tag{7}$$

where again we've taken averages over all households in each village to construct the x's and y's. ε_c represents any other changes over time in villages unrelated to observable features.

Supplementary Figure S9 shows results for re-running this simulation 100 times and re-estimating the three regressions each time. Cross-sectional performance mirrors our main results by construction, but performance in predicting changes over time is much worse. The poorer temporal performance is because the change in y is small relative to the cross-sectional variation in y, which again mirrors what we observe in our wealth data. In essence, the noise in the cross section (i.e. ϵ_{ic1} and ϵ_{ic2}, or factors related to y that are not related to observed features) are diluting any signal in the dy's.

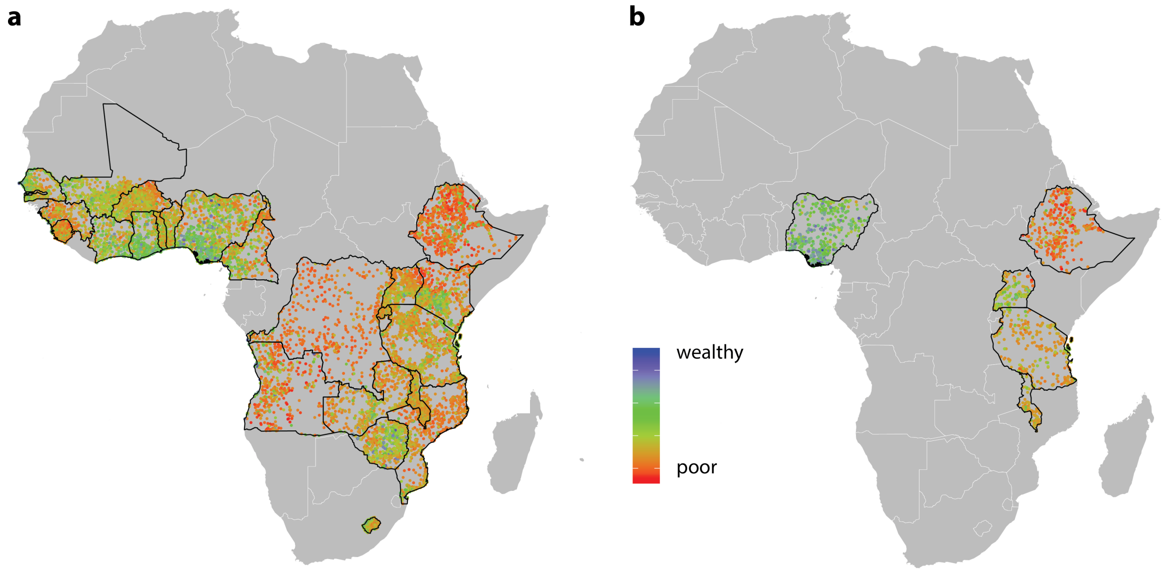

Supplementary Figure 1: Geographic locations of survey clusters, colored by wealth index. **a** DHS clusters. **b** LSMS clusters.

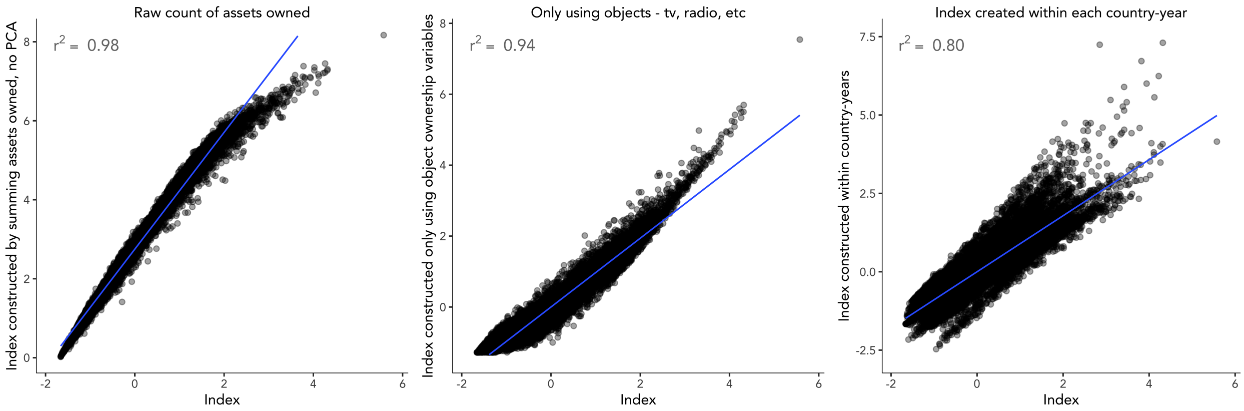

Supplementary Figure 2: **Asset wealth index is robust to alternative methods for constructing it**. Each plot compares baseline index contructed from PCA on pooled households across all countries, against alternate measures of constructing index; r^2 for each comparison shown in upper left of each plot. Left plot: baseline index vs index constructed by simply summing the number of owned assets in each household. Middle plot: baseline index vs PCA index constructed only from owned objects and not home attributes (roof type, wall type, etc). Right plot: baseline index vs PCA index constructed separately for each country-year.

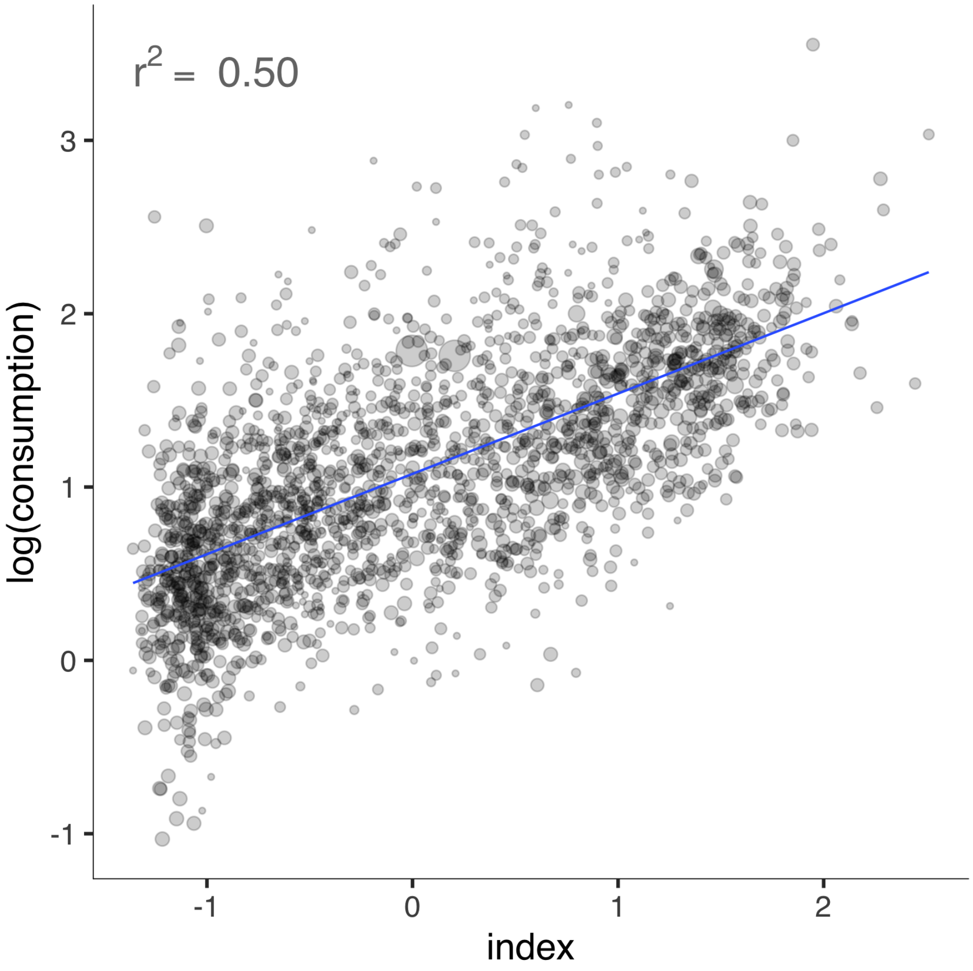

Supplementary Figure 3: **Relationship between consumption and assets at the village level**. Each dot is a village in LSMS sized by number of households surveyed, comparing village-average asset wealth (x-axis) and log average per capita consumption expenditure in the same village (y-axis). The weighted r^2 of a regression of log consumption on assets is 0.5. Data are pooled from LSMS surveys from three countries: Nigeria, Ethiopia, and Tanzania.

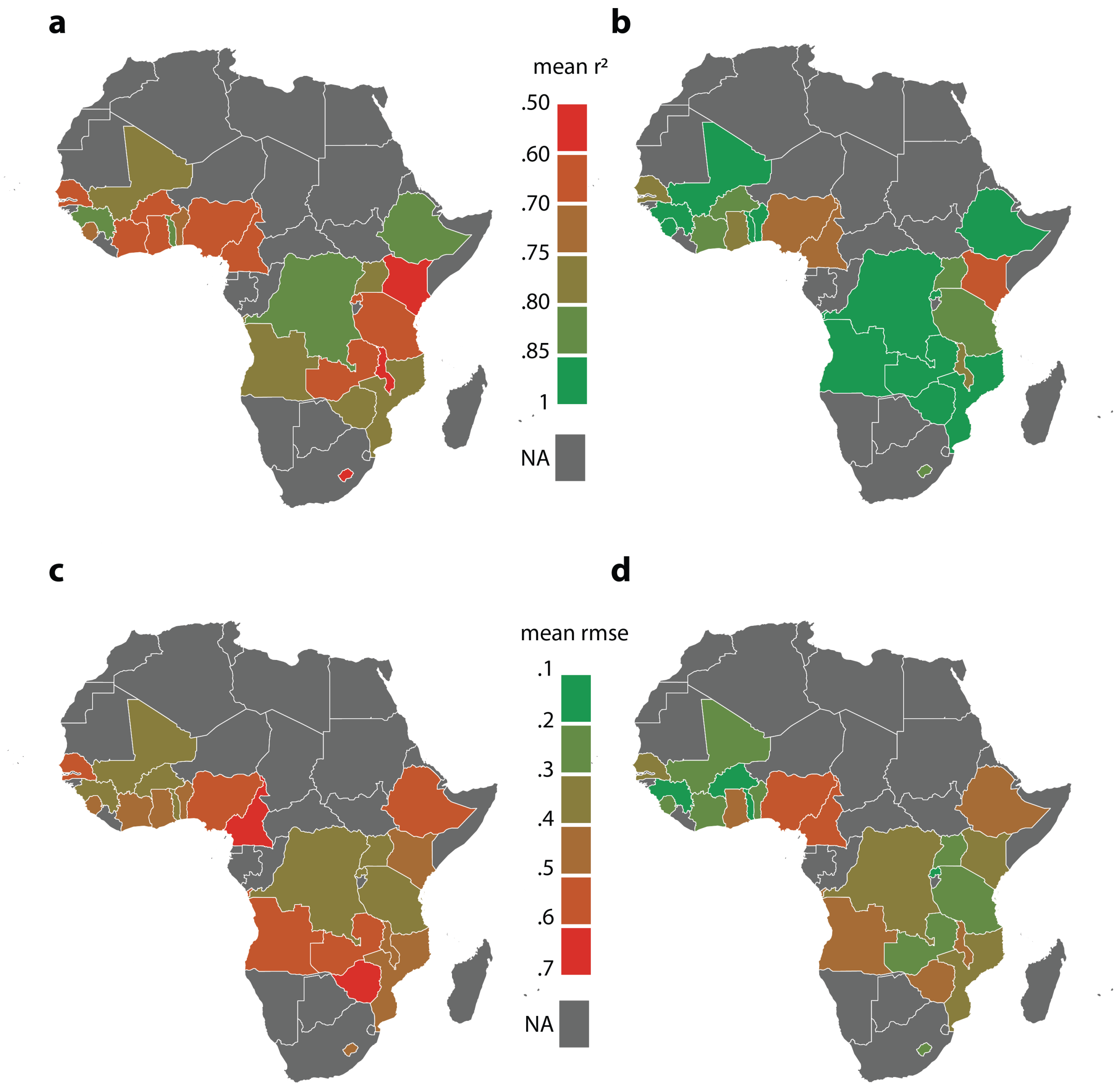

Supplementary Figure 4: **Comparison between country level RMSE and country level** r^2. **a** Village level r^2. **b** District level r^2. **c** Village level RMSE. **d** District level RMSE. The pooled sample of predictions at a village level has an RMSE of 0.46. The pooled sample of predictions at a district level has an RMSE of 0.28, which is similar to the RMSE between census and DHS observations at the district level (0.26). The underlying DHS pooled wealth index at the household level has a mean equal to 0 and standard deviation of 1 by construction, so RMSE values can be interpreted in standard deviations of the wealth index.

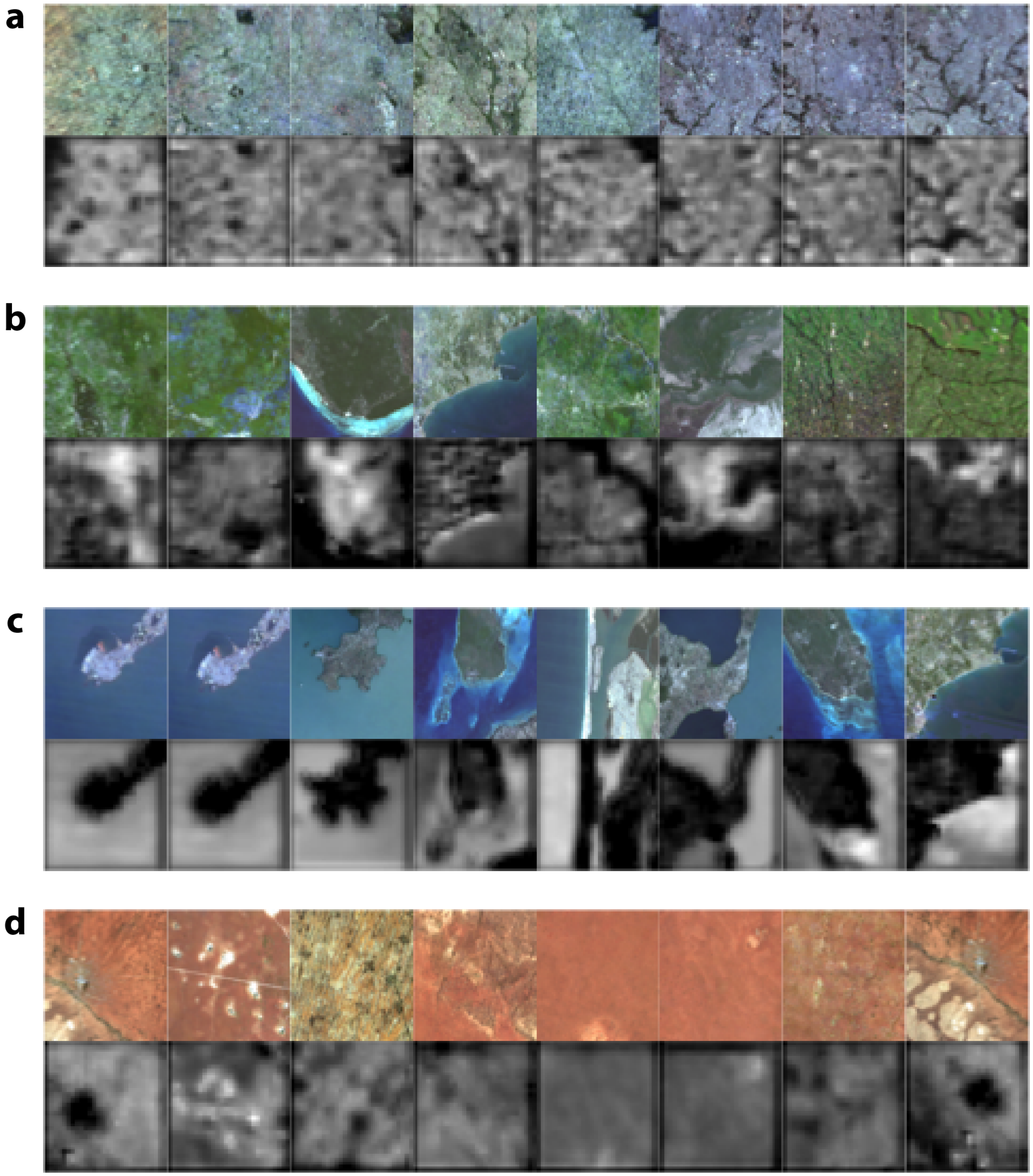

Supplementary Figure 5: **Visualization of maximally activating images for four selected filters**. Each row pair shows the original Landsat input image on the top and the corresponding activation map for a given filter from the in-country `CNN MS+NL` model on the bottom. Selected filters appear to activate in the presence of **a** urban areas, **b** farmland or greenery, **c** water bodies, and **d** desert terrain.

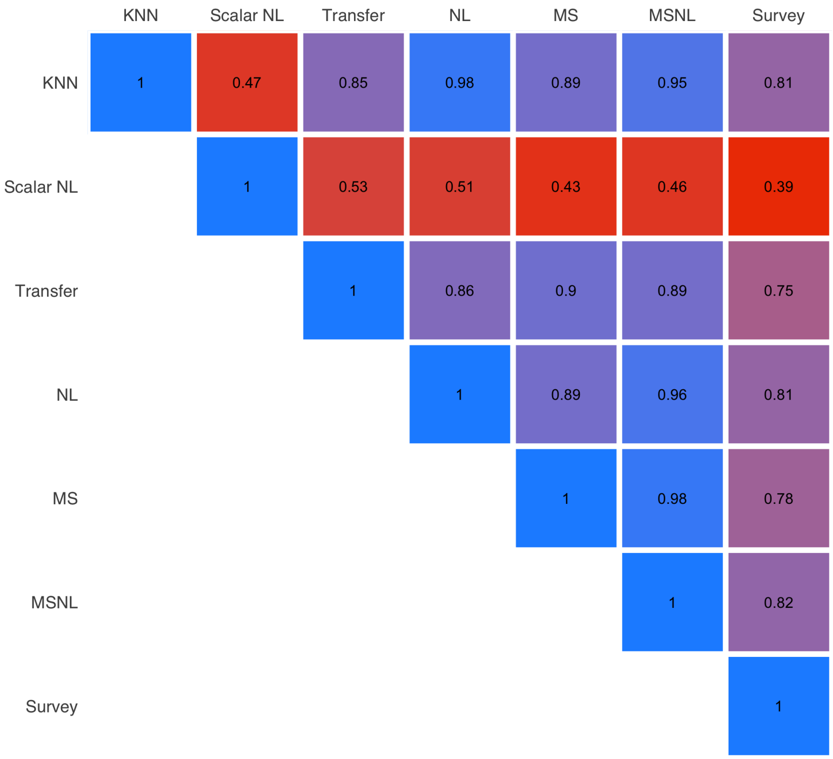

Supplementary Figure 6: **Correlation of cluster-level wealth predictions in held-out countries**, for the models shown in Fig. 3a, as well as the ground-based measurements (labeled *Survey*). *MS, NL, MSNL*, and *Transfer* refer to CNN models as described in the text. More blue-ish colors correspond to higher correlation, and red-ish colors to lower correlation.

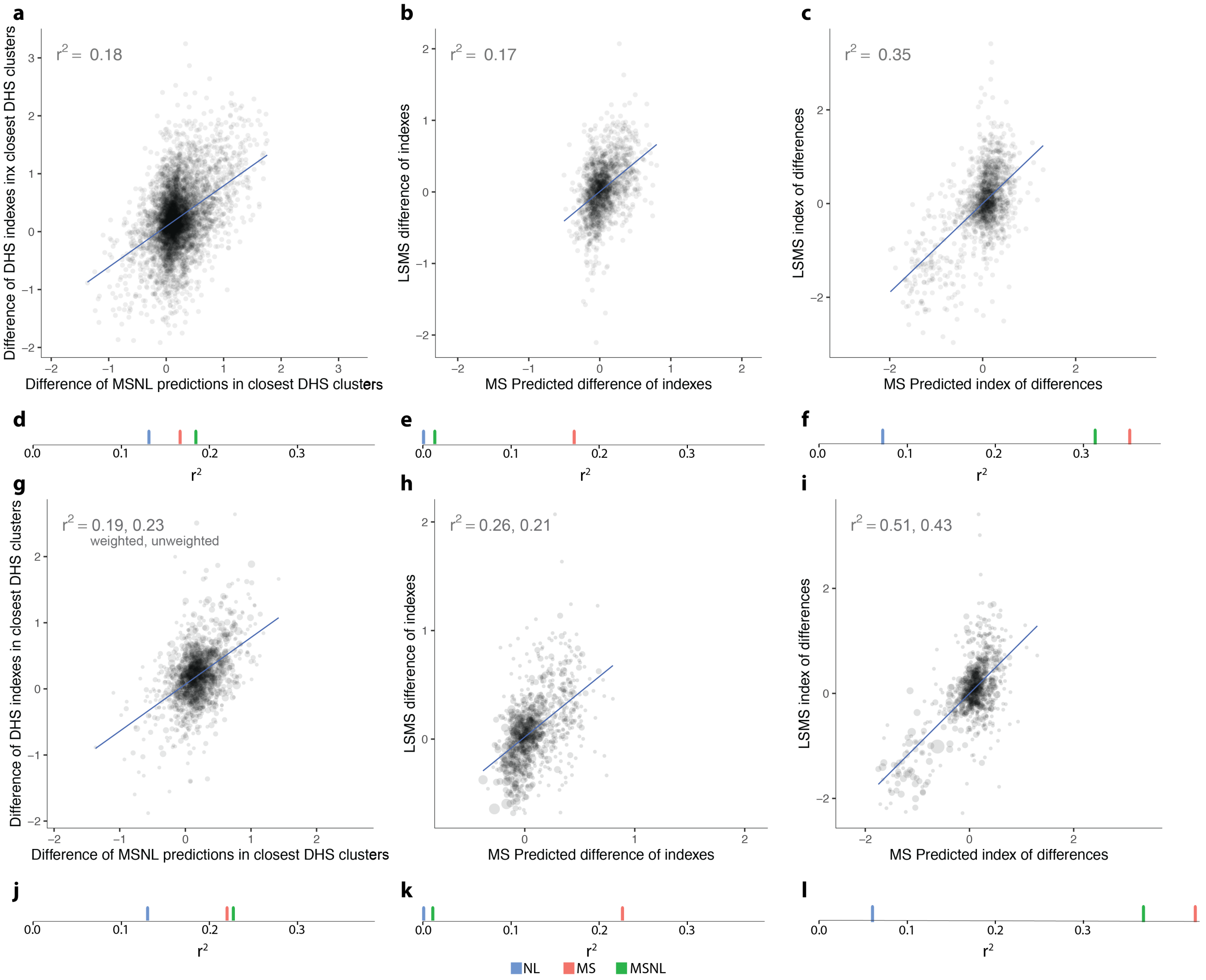

Supplementary Figure 7: **Satellite predictions of ground-measured changes in wealth over time, for different ways of calculating changes**. **a** Assuming that clusters close to each other are more similar than those further apart, we match each DHS cluster (roughly, village) to its nearest geographic neighbor in the next time period to create a synthetic panel, treating those matches as the same cluster in each time period. Each dot is a set of matched villages, and represents the change in satellite-based wealth predictions over time at those villages, versus the change in the survey-based wealth index over time at the same villages. Clusters are only included if they have a neighbor within 10km. **b** Performance of satellite-based model trained to predict the change in wealth index over time, using village-level panel data from the Living Standards Measurement Surveys (LSMS). **c** Same as Fig 4a, where we compute the change in each assets over time and compute an index of those changes. Plot shows performance of model trained to predict this index of changes. **d-f** Cross-validated r^2 of models trained on multispectral (MS, red), nightlights (NL, blue), and both (MSNL, green). **g-i** Same as a-c, but with observations aggregated to the district level. Dot size represents number of village observations in each district, and r^2 is reported both weighted and unweighted by number of villages. **j-l** Same as d-f, but with the unweighted aggregated r^2 for each model.

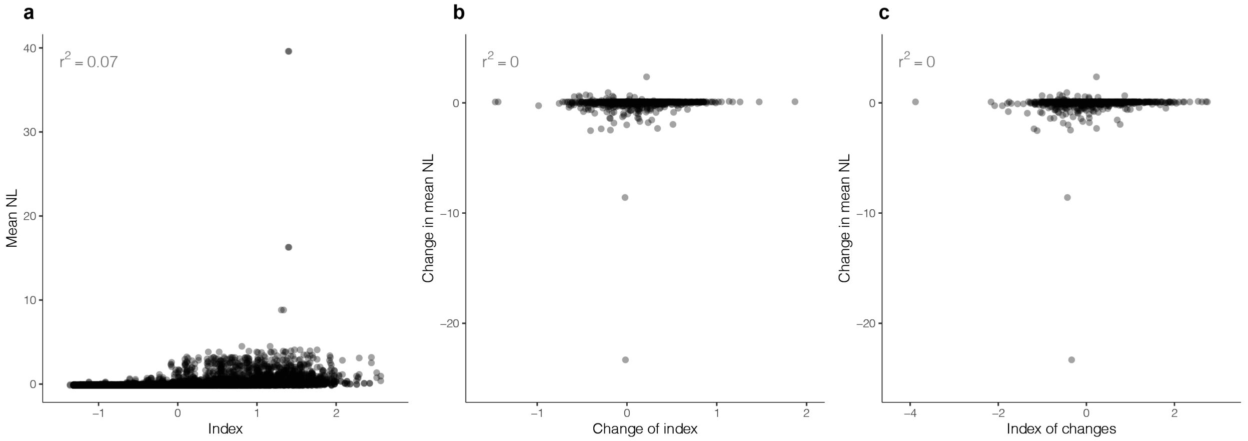

Supplementary Figure 8: **Changes in mean NL are not predictive of changes in wealth index**. **a** Mean NL and the cluster wealth index have an r^2 of 0.19 (estimates weighted by number of households in each cluster). **b** Change in mean NL and the change in wealth are not related. **c** Change in mean NL and the index constructed over changes in asset ownership are not related.

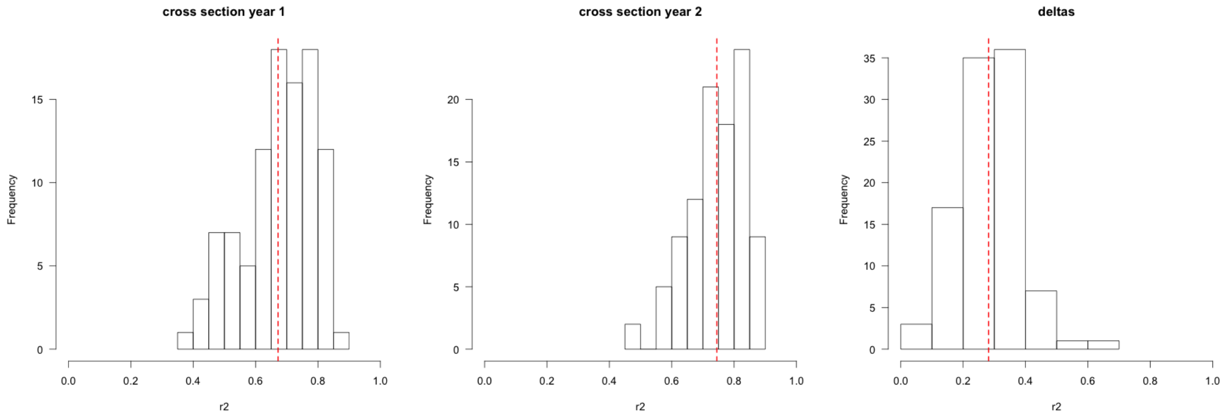

Supplementary Figure 9: **Results from simulation predicting cross sectional and over-time relationships**. First two columns show r^2 from estimating cross-sectional Equations (5) and (6). Third column estimates changes over time from Equation (7). Histograms show results across 100 bootstraps of the simulation described in Methods.

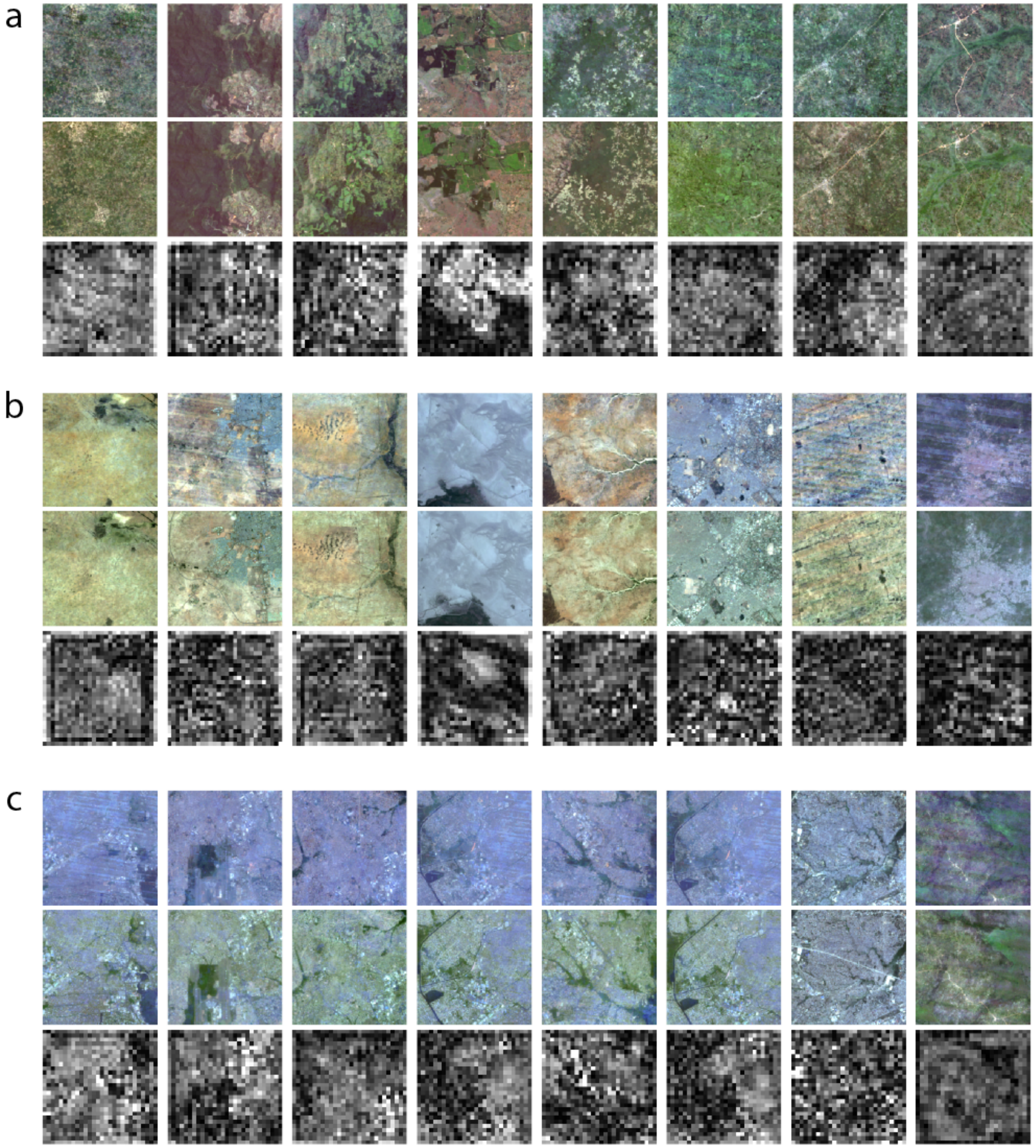

Supplementary Figure 10: **Visualization of maximally activating images for three selected filters from the over-time predictions in Figure 4**. Each triplet of rows shows the original Landsat input image in the base year, the end year, and the corresponding activation map from the `CNN MS` model. Selected filters appear to activate in the presence of **a** farmland or greenery, and **b-c** urban areas.

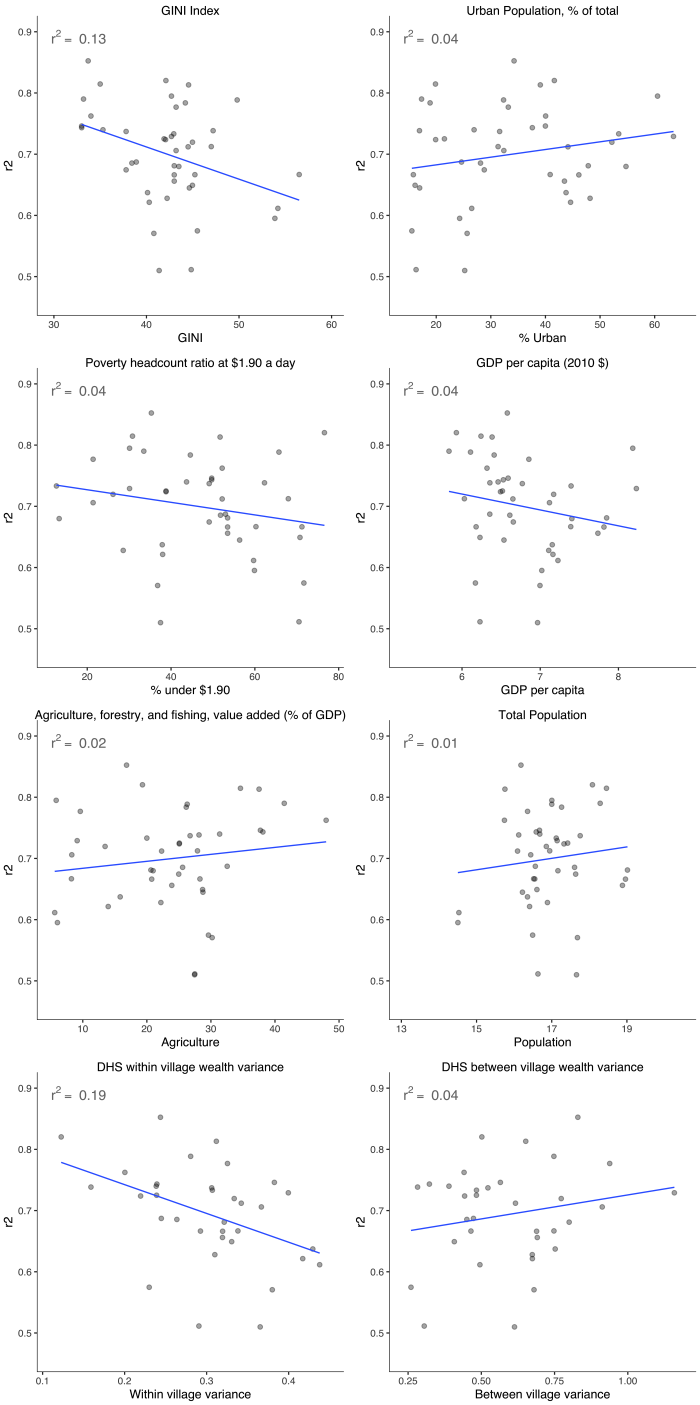

Supplementary Figure 11: **Correlates of predictive performance at the country level**. We relate predictive performance at the country-year level (shown in Fig 2c and 3a) to country-level statistics on inequality (as measured by the gini index), % of population in urban areas, headcount poverty (% of population living under $1.90/day), GDP per capita (log 2010 $), % income from agriculture, and total population, as derived from the World Development Indicators.[37] Additionally, we relate performance to two measures of the variance of wealth from the DHS survey data: the within-village variation in wealth and the between-village variation in wealth. The model performs better in locations with low variance within villages (clusters), and in locations with higher variance between villages.

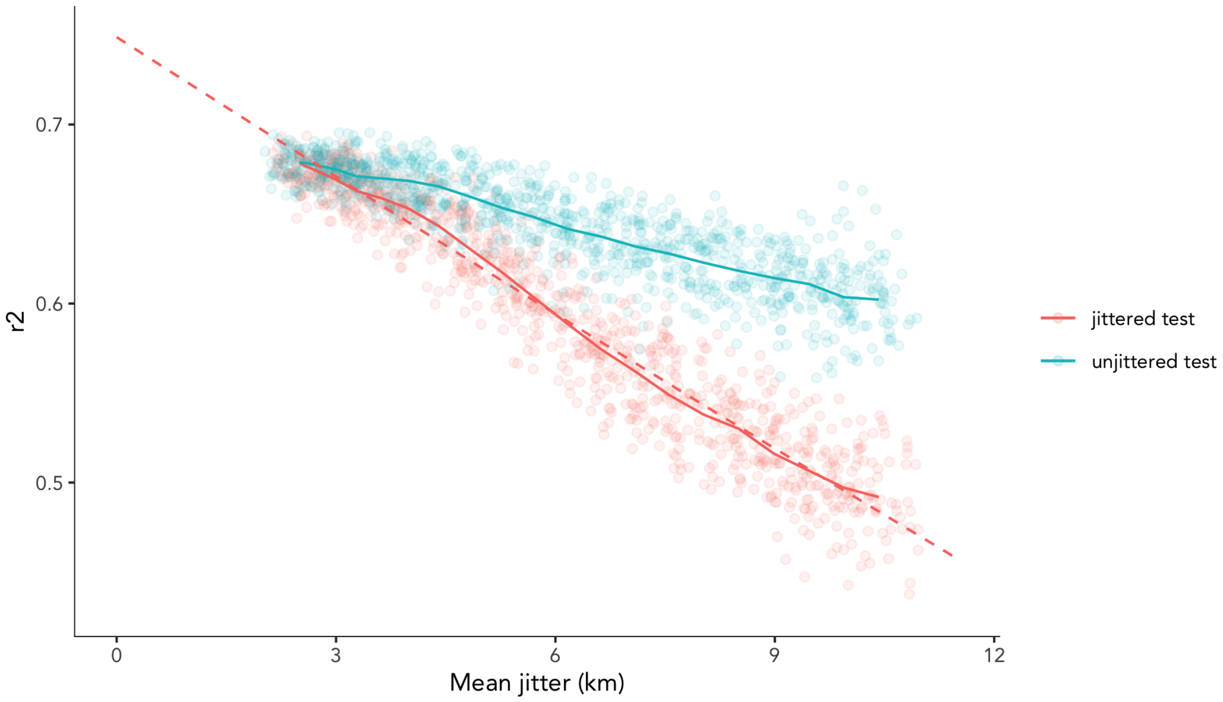

Supplementary Figure 12: **Effect of noise in village locations on model performance**. To understand the impact of existing noise in village locations on model performance (noise that was purposely added by survey implementers to protect privacy), we add additional jitter to each survey location in the training set or both the train and test sets and re-evaluated model performance. We assume baseline jitter is 2.5km in our data, as DHS reports jitter is uniformly distributed between 0-5km.[25] Each dot is an individual experiment; to ease computation, these experiments are run on the NL KNN model, which performed nearly as well as our deep learning model in predicting spatial variation in wealth. Performance degrades with additional jitter, although much less rapidly when evaluating on unjittered test data (blue) as compared to jittered test data (red). This suggests that the true (unobserved) performance of our models is already higher than what our jittered test data would suggest. Using these results to extrapolate backward to a setting of no noise (dotted red line) suggests that locational noise in ground data is reducing model performance by $r^2 = 0.07$.

Supplementary Table 1: DHS Surveys

Country	Year	# villages	# households	# villages used	# of households used
Angola	2011	238	8021	230	7744
Angola	2015	625	15057	625	15057
Benin	2012	750	17395	746	17305
Burkina Faso	2010	573	14326	541	13521
Burkina Faso	2014	252	6316	248	6214
Cameroon	2011	578	13957	576	13910
Côte d'Ivoire	2012	351	9391	341	9101
Democratic Republic of Congo	2013	536	18004	492	16534
Ethiopia	2010	596	16509	571	15852
Ethiopia	2016	643	16480	622	15988
Ghana	2014	427	11726	422	11578
Ghana	2016	200	5774	192	5536
Guinea	2012	300	7060	300	7060
Kenya	2014	1594	35955	1585	35751
Kenya	2015	245	6445	245	6445
Lesotho	2009	400	9281	395	9166
Lesotho	2014	399	9311	399	9311
Malawi	2010	849	24689	827	24075
Malawi	2012	140	3402	140	3402
Malawi	2014	140	3403	140	3403
Malawi	2015	850	26310	850	26310
Mali	2012	413	10085	413	10085
Mali	2015	177	4239	177	4239
Mozambique	2009	270	6036	270	6036
Mozambique	2011	610	13860	609	13840
Nigeria	2010	239	5871	239	5871
Nigeria	2013	896	38337	889	38032
Nigeria	2015	326	7724	322	7629
Rwanda	2010	492	12476	492	12476
Rwanda	2014	492	12624	492	12624
Senegal	2010	391	7889	385	7767
Senegal	2012	200	4172	200	4172
Sierra Leone	2013	435	12536	435	12536
Tanzania	2010	475	9586	458	9248
Tanzania	2011	583	9993	573	9815
Tanzania	2015	608	12499	608	12499
Togo	2013	330	9418	330	9418
Uganda	2009	170	4410	170	4410
Uganda	2011	404	8992	400	8898
Uganda	2014	210	5118	208	5067
Zambia	2013	721	15669	719	15624
Zimbabwe	2010	406	9697	393	9384
Zimbabwe	2015	400	10417	400	10417
Total		22,143	569,175	19,699	503,350

Supplementary Table 2: Folds used for DHS Out-of-Country Training.

Fold	Countries	Village Count
A	Angola, Côte d'Ivoire, Ethiopia, Mali, Rwanda	3963
B	Benin, Burkina Faso, Guinea, Sierra Leone, Tanzania	3909
C	Cameroon, Ghana, Malawi, Zimbabwe	3940
D	Democratic Republic of Congo, Mozambique, Nigeria, Togo, Uganda	3929
E	Kenya, Lesotho, Senegal, Zambia	3928

Supplementary Table 3: Split of the 5 folds used for all cross-validated training.

Train	Val	Test
C, D, E	B	A
A, D, E	C	B
A, B, E	D	C
A, B, C	E	D
B, C, D	A	E

Supplementary Table 4: Loadings on individual household assets from first principal component

Asset	Loading
water	0.50
toilet	0.61
floor	0.70
electricity	0.80
radio	0.48
tv	0.82
fridge	0.68
motorbike	0.48
car	0.59
phone	0.25
rooms per person	0.15
