# Supplementary material for: Using publicly available satellite imagery and deep learning to understand economic well-being in Africa
Source: Nat Commun. 2020 May 22;11:2583. doi: 10.1038/s41467-020-16185-w (PMC7244551; doi:10.1038/s41467-020-16185-w)
Supplement: Supplementary file 3 — Reporting Summary [file 41467_2020_16185_MOESM3_ESM.pdf]

nature research

Corresponding author(s): Marshall Burke

Last updated by author(s): Apr 7, 2020

Reporting Summary

Nature Research wishes to improve the reproducibility of the work that we publish. This form provides structure for consistency and transparency in reporting. For further information on Nature Research policies, see Authors & Referees and the Editorial Policy Checklist.

Statistics

For all statistical analyses, confirm that the following items are present in the figure legend, table legend, main text, or Methods section.

n/a	Confirmed	
☐	☒	The exact sample size (n) for each experimental group/condition, given as a discrete number and unit of measurement
☐	☒	A statement on whether measurements were taken from distinct samples or whether the same sample was measured repeatedly
☐	☒	The statistical test(s) used AND whether they are one- or two-sided<br>*Only common tests should be described solely by name; describe more complex techniques in the Methods section.*
☐	☒	A description of all covariates tested
☐	☒	A description of any assumptions or corrections, such as tests of normality and adjustment for multiple comparisons
☐	☒	A full description of the statistical parameters including central tendency (e.g. means) or other basic estimates (e.g. regression coefficient) AND variation (e.g. standard deviation) or associated estimates of uncertainty (e.g. confidence intervals)
☐	☒	For null hypothesis testing, the test statistic (e.g. F, t, r) with confidence intervals, effect sizes, degrees of freedom and P value noted<br>*Give P values as exact values whenever suitable.*
☒	☐	For Bayesian analysis, information on the choice of priors and Markov chain Monte Carlo settings
☒	☐	For hierarchical and complex designs, identification of the appropriate level for tests and full reporting of outcomes
☐	☒	Estimates of effect sizes (e.g. Cohen's d, Pearson's r), indicating how they were calculated

Our web collection on statistics for biologists contains articles on many of the points above.

Software and code

Policy information about availability of computer code

Data collection	The data we use were collected by others, either as household survey data or satellite data.
Data analysis	Javascript on google earth engine was used to process imagery; python was used to construct and train the CNN; R was used to assemble the household data and for most figure generation. All code is available in the github repo linked in our paper.

For manuscripts utilizing custom algorithms or software that are central to the research but not yet described in published literature, software must be made available to editors/reviewers. We strongly encourage code deposition in a community repository (e.g. GitHub). See the Nature Research guidelines for submitting code & software for further information.

Data

Policy information about availability of data

All manuscripts must include a data availability statement. This statement should provide the following information, where applicable:

- Accession codes, unique identifiers, or web links for publicly available datasets
- A list of figures that have associated raw data
- A description of any restrictions on data availability

All data needed to replicate our results are available at the Github repo linked in our paper.

Field-specific reporting

Please select the one below that is the best fit for your research. If you are not sure, read the appropriate sections before making your selection.

☐ Life sciences ☐ Behavioural & social sciences ☒ Ecological, evolutionary & environmental sciences

Ecological, evolutionary & environmental sciences study design

All studies must disclose on these points even when the disclosure is negative.

Study description	Quantitative analysis of whether household survey data (aggregated to village level) can be predicted by satellite imagery.
Research sample	19,700 villages across Sub-saharan Africa, as measured in publicly-available household survey data from the Demographic and Health Surveys.
Sampling strategy	DHS follow a two-stage sampling strategy, first sampling villages (proportional to population) and then randomly sampling households within villages. We take the average of computed wealth values across households within each village to get a village-level wealth estimate.
Data collection	the DHS collected the survey data. We use data since 2006, and all data were collected as a collaboration between DHS and country statistical offices.
Timing and spatial scale	We use data collected between 2006 and 2016, across 43 surveys in 23 African countries. Survey data in each country are nationally representative and span the entire country.
Data exclusions	We dropped any observations with missing or erroneous GPS data (i.e. locational data); for the latter, a small number of villages had (0,0) as the location, which is in the middle of the Atlantic Ocean.
Reproducibility	Key analyses were replicated multiple times by more than one researcher on each team. All data and experiments will also be available for public replication upon publication.
Randomization	Our study is observational, with the goal of predicting household survey data from satellites.
Blinding	Our study was blinded in the sense that all our evaluation criteria were computed on data which the model was not trained on (which is standard practice in computer science). That is, our model was blinded to the data on which it would be evaluated.

Did the study involve field work? ☐ Yes ☒ No

Reporting for specific materials, systems and methods

We require information from authors about some types of materials, experimental systems and methods used in many studies. Here, indicate whether each material, system or method listed is relevant to your study. If you are not sure if a list item applies to your research, read the appropriate section before selecting a response.

Materials & experimental systems

n/a	Involved in the study	
☒	☐	Antibodies
☒	☐	Eukaryotic cell lines
☒	☐	Palaeontology
☒	☐	Animals and other organisms
☒	☐	Human research participants
☒	☐	Clinical data

Methods

n/a	Involved in the study	
☒	☐	ChIP-seq
☒	☐	Flow cytometry
☒	☐	MRI-based neuroimaging
